# Fabrication and characterization of a full-size ultra-precise lamellar grating for the Cosmic beamline at ALS-U

**DOI:** 10.1107/S1600577525005946

**Published:** 2025-08-18

**Authors:** Dmitriy Voronov, Sooyeon Park, Lei Huang, Antoine Islegen-Wojdyla, Eric Gullikson, Howard Padmore, Tianyi Wang, Mourad Idir

**Affiliations:** a Advanced Light Source Lawrence Berkeley National Laboratory 1 Cyclotron Rd, MS 2R400 Berkeley CA94720 USA; b NSLS II Brookhaven National Laboratory BNL Building 703 Upton NY11973 USA; c CXRO Lawrence Berkeley National Laboratory 1 Cyclotron Rd, MS 2R400 Berkeley CA94720 USA; RIKEN SPring-8 Center, Japan

**Keywords:** X-ray diffraction grating, groove density, variable line spacing, diffraction efficiency

## Abstract

We have developed a new process for the production of ultra-precise variable line spacing (VLS) lamellar diffraction gratings through nanofabrication. The process enables the fabrication of full-size X-ray gratings with sub-nanometre accuracy in groove depth, an optimal land-to-groove ratio, and uniform groove depth across the entire grating area. We also established a method for evaluating VLS groove density variation using stitched Fizeau interferometry. The measurements confirmed the exceptionally high accuracy of the VLS groove density in the fabricated gratings, which is well within the specification tolerances while the residual groove density errors are vanishingly small. The gold-coated grating demonstrated near-theoretical diffraction efficiency across the energy range of 100–1200 eV.

## Abstract 2

Nanofabrication enables extremely precise and highly efficient variable line spacing diffraction gratings for X-rays.

## 1. Introduction

We have developed a range of techniques over the last years to make near perfect blazed diffraction gratings (Voronov *et al.*, 2016*a* ▸; Voronov *et al.*, 2016*b* ▸; Voronov *et al.*, 2018 ▸; Voronov *et al.*, 2022 ▸; Voronov *et al.*, 2023*a* ▸) for soft and tender X-rays. These provide the ultimate in terms of diffraction efficiency for grazing incidence optics. We have also extended this work using multilayer coatings to provide higher resolution and efficiency in an extended angular range (Voronov *et al.*, 2011 ▸; Voronov *et al.*, 2014 ▸; Voronov *et al.*, 2016*c* ▸; Voronov *et al.*, 2021 ▸). However, for some cases laminar gratings provide adequate performance, within a factor of two of an optimum blazed grating, with much lower fabrication costs and can provide very useful suppression of harmonics and extremely low scattered light. They also provide a less peaked diffraction efficiency curve than a blazed grating, in a constant deviation angle geometry.

The COSMIC beamline at the Advanced Light Source (ALS), USA, for coherent imaging and scattering (Shapiro *et al.*, 2013 ▸; Shapiro *et al.*, 2018 ▸) will be replaced as part of the upgrade of the ALS, ALS-U. The new beamline utilizes the existing undulator, but the optical system will be replaced by an entrance slitless variable-included-angle, plane variable-line-spacing (VLS) grating monochromator (Harada *et al.*, 1984 ▸; Itou *et al.*, 1989 ▸; Reininger, 2011 ▸) together with an adaptive focusing system in the non-dispersive direction. This beamline will be equipped with two blazed gratings for the higher energy ranges where harmonics are less of an issue, and the lamellar grating described here will be used at the low energy end of the monochromator energy range, where high harmonic suppression is essential.

VLS gratings are used in many types of X-ray monochromators and spectrographs (Underwood & Koch, 1997 ▸; Reininger & Castro, 2005 ▸; Strocov *et al.*, 2011 ▸; Warwick *et al.*, 2014 ▸; Chuang *et al.*, 2016 ▸) due to their superior imaging performance over straight ruled gratings in classical geometries. The groove density, *g*(*x*), of a VLS grating varies along the grating length coordinate, *x*, according to a polynomial law,
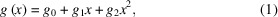
where *g*_0_, *g*_1_ and *g*_2_ are polynomial coefficients. Lamellar VLS grating patterns are normally made by holography. This method uses a laser with a long longitudinal coherence length (typically an ion laser) together with mirror and lens optics to create two beams that interfere on a photoresist coated substrate producing the required groove spacing variation. However, as VLS gratings require highly precise groove placement to enable focusing and aberration correction at the level required, the inevitable imperfections in the custom holographic optics and their alignment often lead to unacceptably large errors. To address the groove placement accuracy problem, we used electron beam lithography (EBL) to record the grating pattern. This technique offers nanometre-scale groove placement accuracy over large areas and provides an essentially perfect grating pattern.

The metrology of VLS gratings is of great importance for both process development and evaluation of the groove density accuracy of a final grating. A few optical tools such as optical diffractometers (Lemke *et al.*, 2022 ▸) and long trace profilers (Cocco *et al.*, 2003 ▸) are currently employed to determine the polynomial coefficients of the VLS groove density. Such measurements provide one-dimensional data along the trace with a spatial resolution of about 1–2 mm defined by the size of the laser spot on the optics surface. A two-dimensional groove density map can be obtained by Fizeau phase shifting interferometry which is used for characterization of diffraction gratings in the Littrow configuration (Palmer, 2020 ▸). This technique provides better resolution due to the smaller pixel size (typically 0.2 mm) of the interferometer camera. Measurements are straightforward for constant groove density gratings which produce a plane diffracted wavefront from the incident plane wave from the interferometer, but this method is not possible in the case of VLS gratings due to the highly curved wavefront which results in dense fringes and as a result strong retrace errors. Due to the limitations of existing techniques, in this work we have developed VLS grating measurements using a stitching procedure developed earlier for non-planar X-ray optics at the NSLS II (Huang *et al.*, 2019 ▸; Huang *et al.*, 2020 ▸). As the interferogram of only a very small section of the grating is measured at any position, essentially limiting image acquisition to one fringe, interferometer errors should be small. Unlike the full aperture technique used for constant line density gratings though, data on the full grating have to be assembled by stitching together data from the small sub-aperture components.

The diffraction efficiency of a lamellar grating maximizes at an optimal groove depth which typically ranges from 5 to 25 nm for soft X-ray gratings, depending on photon energy and the grazing incidence angle. In order to guarantee that the maximum efficiency is achieved close to the design value, this requires sub-nanometre control over the groove depth. Typically used groove shaping methods such as ion beam etching or reactive plasma etch rely entirely on thorough calibration of etch rates during preliminary test etching experiments since on-the-fly monitoring of the groove depth is not available using standard tools. Even a tiny etch rate miscalibration, non-reproducibility or non-uniformity will result in substantial groove depth errors which have a significant impact on the diffraction efficiency and the operational energy range of the grating. In this work we have developed an alternative groove etch process which allows groove depth monitoring and provides sub-nanometre accuracy of the groove depth as well as perfect groove depth uniformity over the grating area.

We demonstrate the process feasibility by making a prototype of a VLS lamellar grating for the Cosmic beamline of the ALS-U. The parameters of the grating and specification tolerances are listed in the Table 1 ▸. The tolerances for groove parameters and especially for the polynomial terms of the VLS groove density are fairly relaxed and were dictated by limitations of the conventional fabrication methods originally to be used rather than the optical design requirements. The new fabrication process described in this paper provides much higher accuracy and can meet much tighter tolerances as will be shown below.

## 2. Experimental

### 2.1. Fabrication

The grating prototypes were made using Si (100) single crystal substrates with dimensions of 200 mm × 40 mm × 20 mm. The substrates were polished flat down to λ/10 flatness which is good enough for process development purposes. To meet the X-ray optics tolerances, the surface could be brought to a sub-nanometre flatness by use of a post-ruling ion beam figuring process as was reported earlier (Voronov *et al.*, 2023*b* ▸; Voronov *et al.*, 2024 ▸).

We made two almost identical lamellar gratings with different groove depth. Grating #1 had a groove depth goal of 19 nm for the soft X-ray COSMIC grating, while grating #2 with grooves of an increased depth of 40 nm was used for groove density measurements using Fizeau interferometry. The deeper grooves of the grating #2 provided higher diffraction efficiency at a wavelength of 632.8 nm of the interferometer, enabling measurements using high diffraction orders.

**Figure 1:**
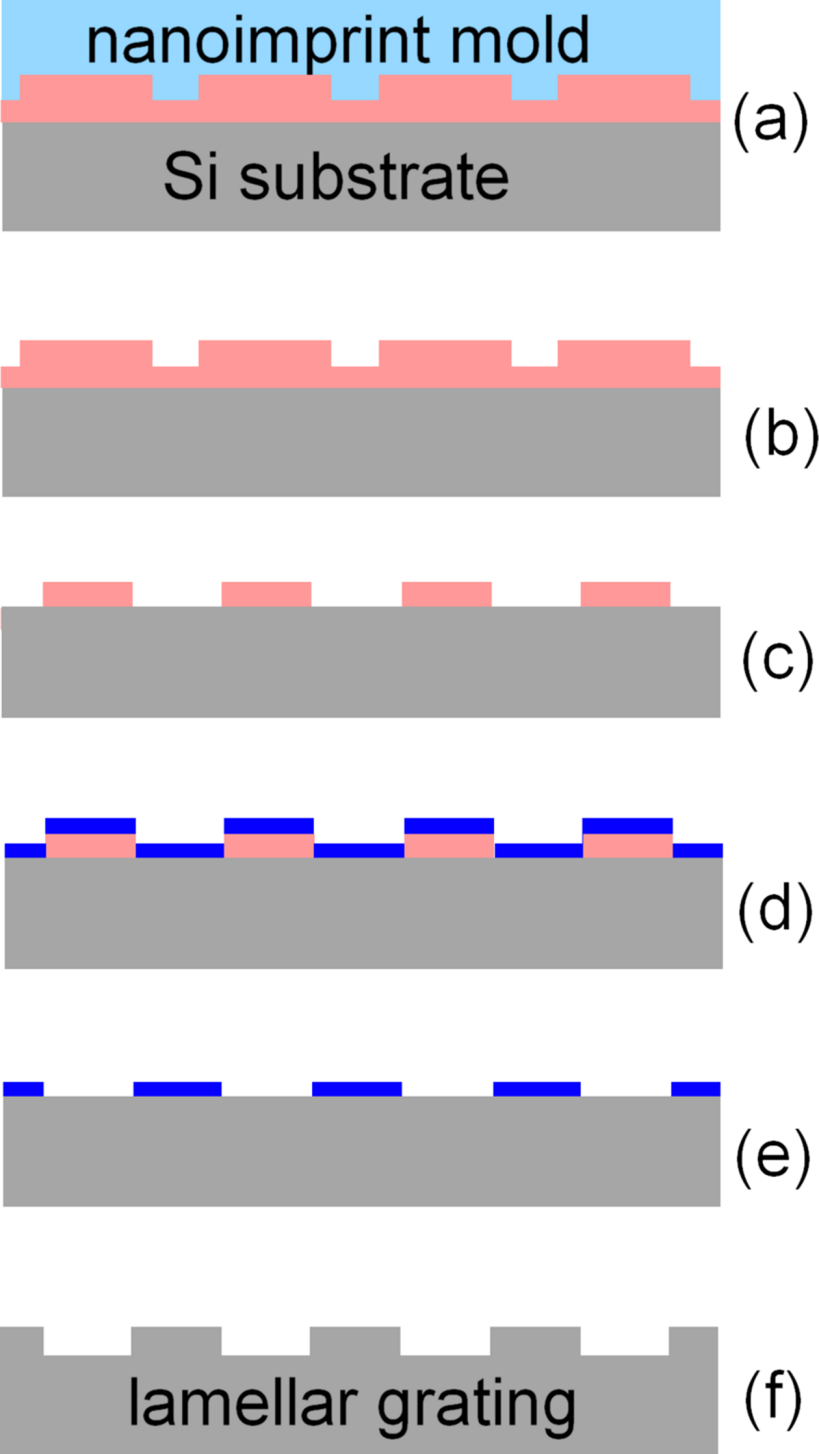
Process flow for lamellar gratings: nanoimprinting (*a*), resist pattern after demolding (*b*) and residual layer etch (*c*), Cr deposition (*d*), hard mask after lift-off (*e*), and the final grating after groove etching and mask removal (*f*). The colors depict Si (gray), quartz (light blue), resist (pink) and Cr (blue).

The fabrication process flow is depicted in Fig. 1 ▸. Electron beam lithography was used to record the 175 mm × 20 mm VLS grating pattern on a 6-inch by 6-inch fused silica plate and transferred into a 70 nm thick Cr layer on top of the quartz surface. In addition to the VLS pattern, two 160 mm × 7 mm supplementary patterns with constant groove density of 178.96 lines mm^−1^, corresponding to the *g*_0_ term of the VLS pattern, were recorded. One of the supplementary patterns was used as a test pattern for measurements of the groove depth in the course of the groove etching process, while the other served as a reference pattern for evaluation of the groove placement accuracy using Fizeau interferometry. All the patterns were recorded with an accuracy specification of 20 nm in a single lithography run, so any possible groove placement errors are expected to be the same for all three patterns. It should be noted that the cost of the EBL process is highly dependent on the required accuracy. In the process we use, we can specify accuracy down to 2 nm over a 6-inch by 6-inch mask for production gratings, but for this development demonstration we used a 20 nm accuracy process due to its much lower cost.

**Figure 2:**
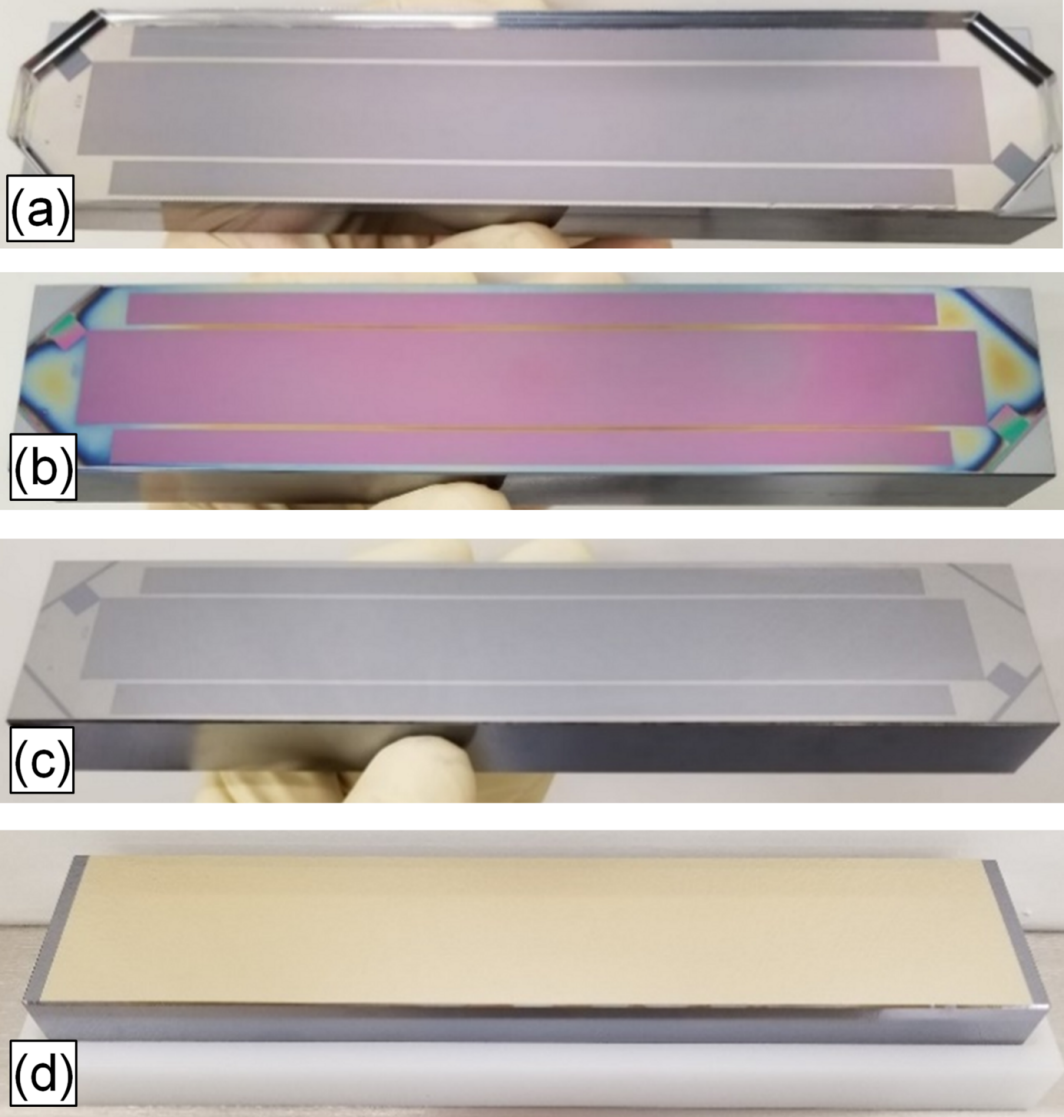
Photographs of the mold/substrate sandwich (*a*), the resist pattern after demolding (*b*), hard mask after lift-off (*c*) and final Au-coated grating (*d*).

**Figure 3:**
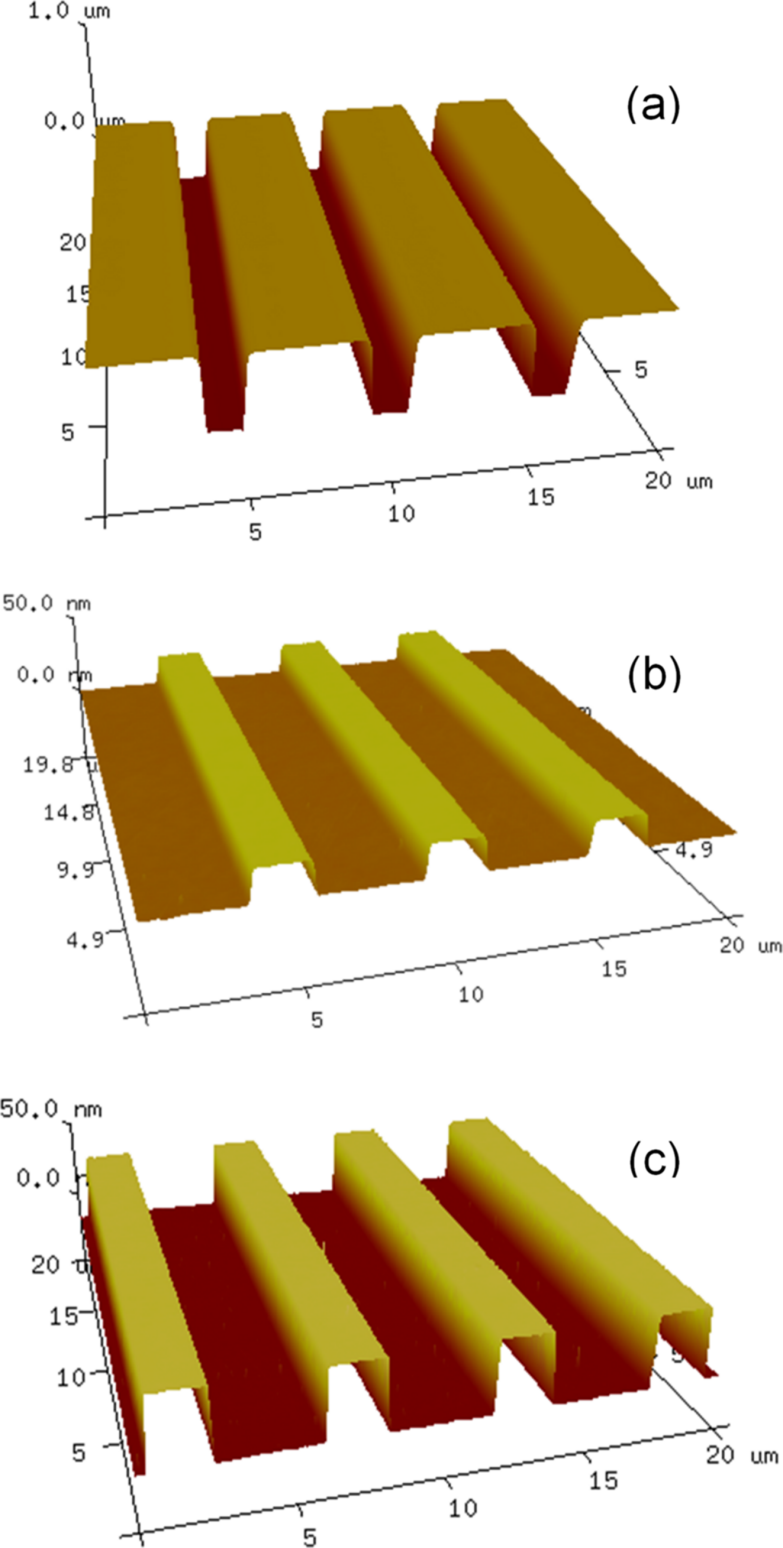
AFM images of the resist pattern after nanoimprint lithography (*a*) and the final Si grating #1 (*b*) and grating #2 (*c*) after RCA etch and mask removal.

A reactive ion etch was used to make 600 nm deep grooves in the quartz plate to make a mold for the following nanoimprinting step. Nanoimprinting is widely used for making a variety of diffraction structures of small or medium size (Schleunitz *et al.*, 2011 ▸). Our large area nanoimprinting process was performed using a UV-curable resist UVCur-26SF. A cushion press setup was used to squeeze the resist between the grating substrate and the mold and then the resist was cured by UV exposure. The mold/substrate sandwich and the resist pattern after the sandwich separation are shown in Figs. 2 ▸(*a*) and 2 ▸(*b*), respectively. An atomic force microscopy (AFM) image of the resist pattern is shown in Fig. 3 ▸(*a*). Residual layer thickness as low as 50 nm was estimated as a difference between the total resist layer thickness measured using a Filmetrix F20 thin film measurement instrument (https://www.filmetrics.com/thickness-measurement/f20) and the groove depth measured by AFM.

Etching through the residual layer of the organic resist was performed using ultraviolet ozone (UVO) etch. We chose this method since it provides perfect selectivity of the etching owing to the zero etch rate of Si. Other methods such as plasma etch have a finite etch selectivity and might result in some over-etch into the Si surface, which introduces some uncertainty in the final groove depth as well as groove depth uniformity. On the contrary, UVO etching leaves the Si surface intact, zeroing the possible over-etch errors. Moreover, one can take advantage of the zero etch rate of the Si substrate to control a land-to-groove ratio via some additional etching. Since the UVO etch is rather isotropic, the resists stripe width can be adjusted by an extended UVO etch to achieve an optimum groove-to-land ratio of the grating.

As soon as the residual layer was etched through and the groove-to-land ratio was close to the optimal one, a lift-off process was applied to make a hard mask for the following groove etching process. A 30 nm thick Cr layer was deposited on top of the resist pattern using an e-beam evaporation setup and then the resist was removed by Piranha solution (H_2_SO_4_ + H_2_O_2_), leaving Cr stripes on the Si substrate surface. A photograph of the grating substrate with the Cr hard mask is shown in Fig. 2 ▸(*c*).

Ion etch or plasma etch processes are traditionally used for making binary grooves of a lamellar grating. A target groove depth is achieved by careful calibration of the etch rate, which is usually quite challenging for thick grating substrates and often compromises the groove depth accuracy. Also it is very challenging to achieve an acceptable etch rate uniformity and at the same time a residue-free etching (Lemke *et al.*, 2014 ▸). To address these and other challenges of the thick grating processing with a plasma etch, we developed an alternative process based on wet isotropic etching. An RCA solution (NH_4_OH + H_2_O_2_) was applied to grating #1 to etch grooves of the required depth of 19 nm. The wet chemical etching gives a low etch rate of about 1 nm min^−1^ and very high uniformity of etching, making the etching process highly controllable. The etching was performed in several consecutive runs with intermediate measurements of the groove depth. To do so the Cr mask was removed from a small patch of the test pattern and direct measurements of Si groove depth were performed locally by AFM. Such a groove depth monitoring procedure relies on the presumed very high uniformity of the etch rate of the chemical etching process, which was confirmed later and is described below.

**Figure 4:**
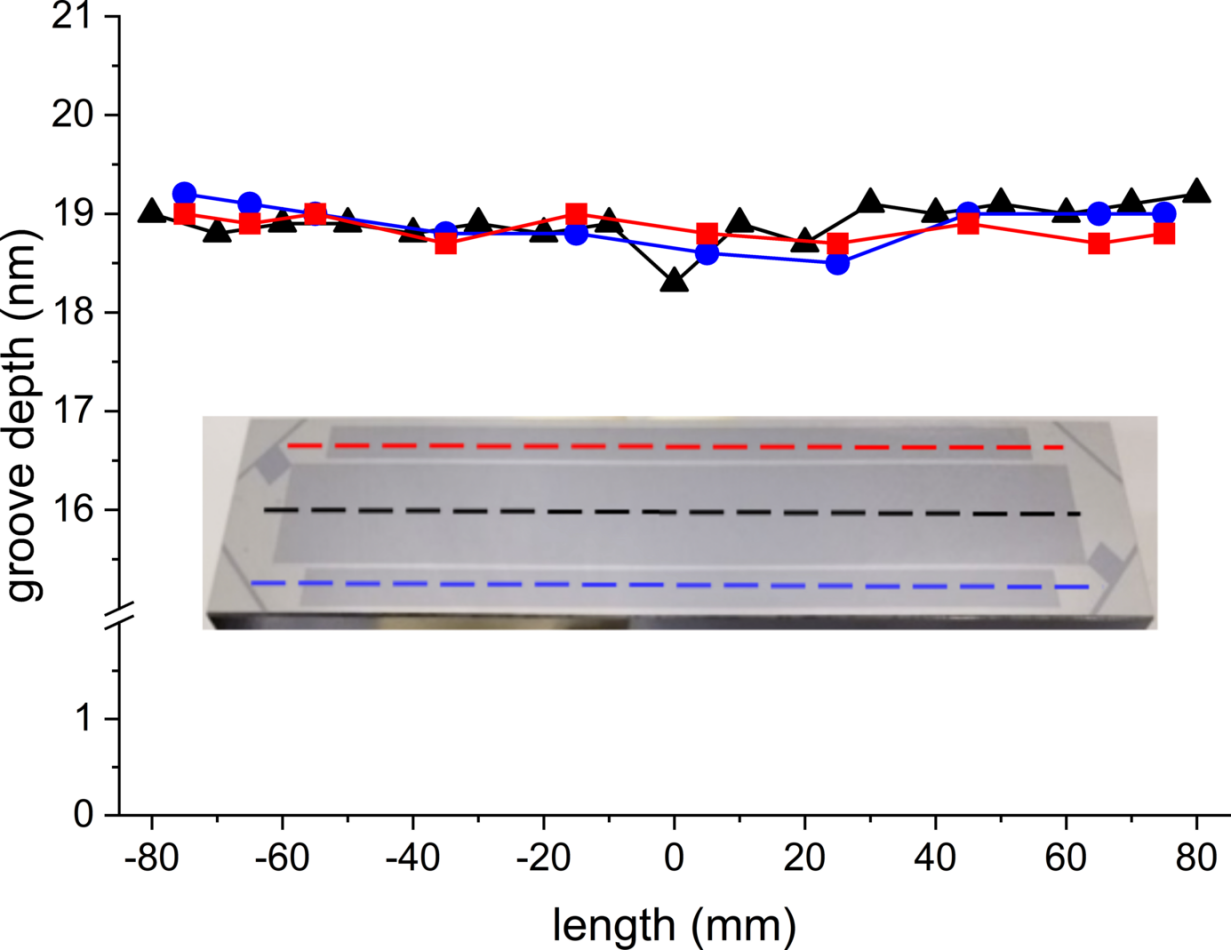
Uniformity of the groove depth measured for the three grating patterns. The measurements were performed along the three traces depicted in the insert by dashed lines.

As soon as the groove depth goal was achieved, the hard mask was stripped off using a 1020 Cr etchant, and the grating was inspected by AFM. The AFM measurements confirmed both the groove depth and the groove-to-land ratio accuracy [Fig. 3 ▸(*b*)], as well as extremely high uniformity of the groove depth measured over the area of all three grating patterns (Fig. 4 ▸). The average groove depth of 18.9 nm with less than 1 nm peak-to-valley variation matches the target value of 19 nm. The measured land-to-pitch ratio of 0.33 is well within the tolerance of the optimal one of 0.38.

Grating #2 was made using a longer wet etching resulting in a groove depth of 40 nm [Fig. 3 ▸(*c*)]. Both gratings were coated with a 30 nm thick Au layer with 5 nm thick Cr bonding layer.

### 2.2. Groove density measurements

The groove density accuracy was evaluated for grating #2 using Fizeau interferometry in the Littrow configuration. The VLS pattern was measured using a stitching procedure developed at NSLS II (Huang *et al.*, 2019 ▸; Huang *et al.*, 2020 ▸). To verify the stitching procedure the reference pattern was measured twice. First, the whole reference pattern was measured in a high diffraction order (with no stitching) and the second measurement was performed for the first diffraction order using the stitching procedure.

#### 2.2.1. Measurements of the reference grating at ALS

**Figure 5:**
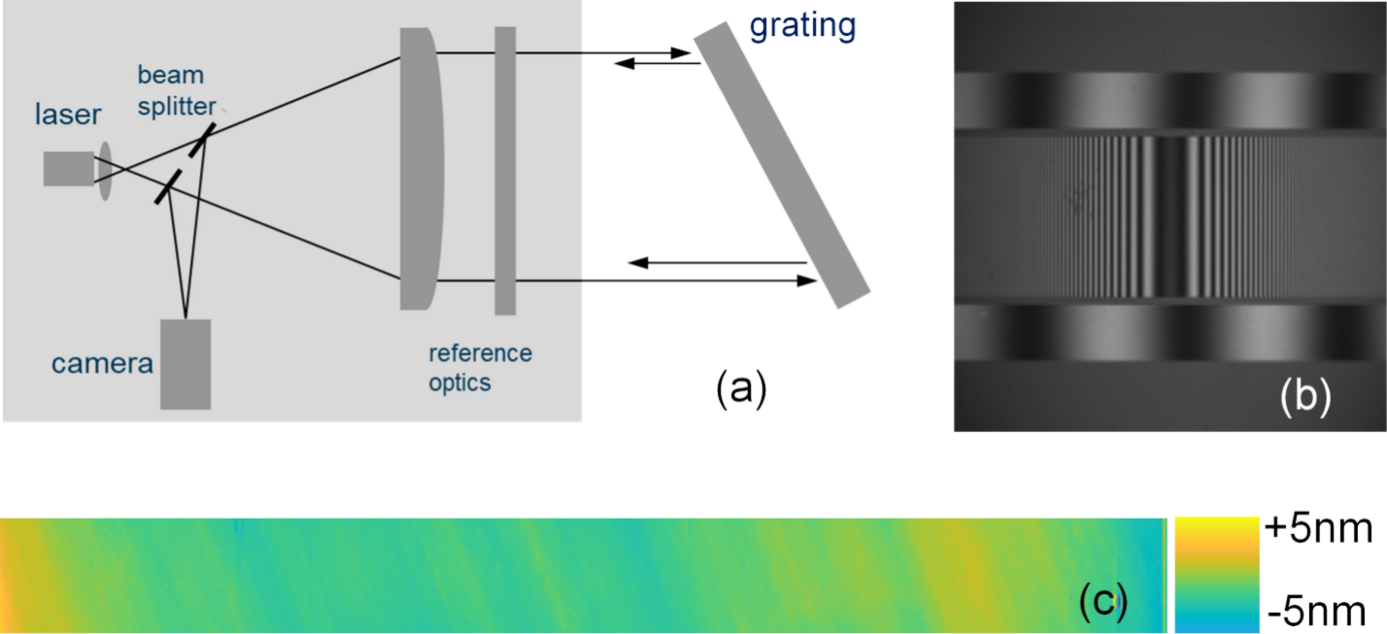
Littrow arrangement (*a*), interference fringes of the reference constant groove density patterns, also showing the VLS pattern in the center (*b*), and differential wavefront error map for the ±1st orders of the reference pattern (*c*).

The high-order measurements of the reference pattern were performed at the ALS using a Zygo Verifire interferometer with a 150 mm diameter reference flat. The procedure and data processing are described elsewhere (Gao & Kimura, 2010 ▸; Voronov *et al.*, 2013 ▸; Gleason *et al.*, 2017 ▸). Briefly, the grating was set at the Littrow angle of 23.35° for the seventh (‘positive’) diffraction order [Fig. 5 ▸(*a*)], so the image of the whole 160 mm long grating was reduced down to 147 mm to fit the 150 mm aperture of the interferometer. After the first measurements the grating was flipped to the ‘negative’ diffraction order arrangement and the second measurements were taken. The difference between the two measurements yields the differential wavefront errors caused solely by the groove density errors while residual substrate surface slope errors are canceled owing to the subtraction.

**Figure 6:**
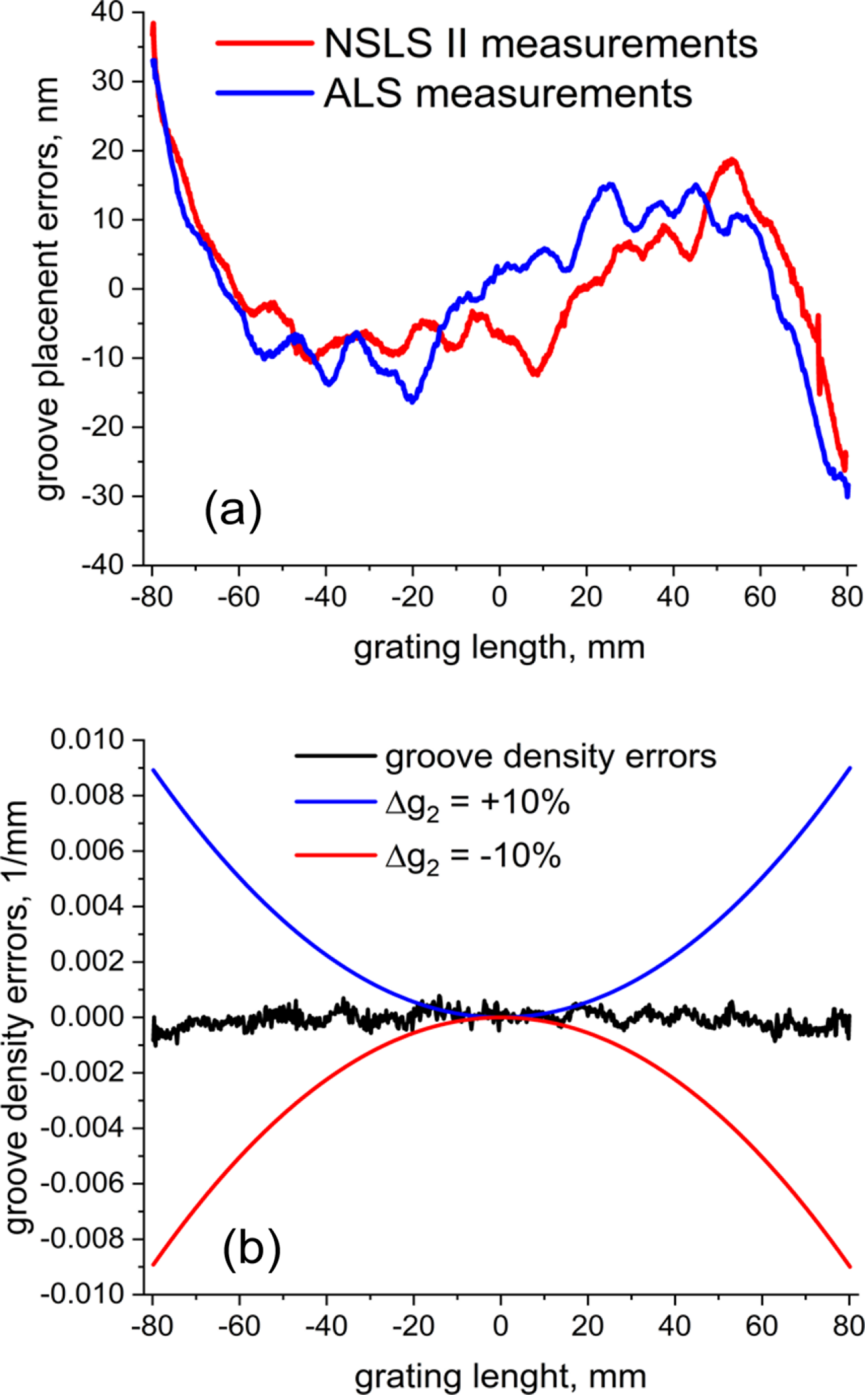
Groove placement errors measured for the ±1st orders (red) and ±7th orders (blue) for the reference grating pattern (*a*). Groove density errors for the reference grating (*b*); the groove density errors caused by ±10% tolerance specified for the *g*_2_ are shown with blue and red curves.

Groove displacement errors, ɛ, *i.e.* the absolute error in a groove position versus an ideal groove position, extracted from the differential wavefront errors, σ, for the *m* the diffraction order by the ratio
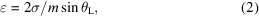
where θ_L_ is the Littrow angle, are shown in Fig. 6 ▸(*a*). The groove displacement does not exceed ±30 nm peak-to-valley which is consistent with the tolerance for the EBL process used. The third polynomial signature of the residual errors is caused by quadratic errors in the groove density, which do not exceed 0.0004 lines mm^−1^ r.m.s. and can be considered as negligibly small. The groove density errors are shown in Fig. 6 ▸(*b*) along with the acceptable groove density variation range caused by ±10% tolerance for the *g*_2_ parameter.

#### 2.2.2. Measurements of the reference grating at NSLS II

The same reference grating pattern was measured in the ±1st order Littrow configuration using a 100 mm aperture Zygo Verifire interferometer at NSLS II. The NSLS II stitching procedure was applied to obtain the wavefront error map from the 160 mm long constant groove density reference pattern [Fig. 5 ▸(*c*)]. The groove placement errors measured by both methods are in excellent agreement as seen in Fig. 6 ▸(*a*). The difference of only 7 nm r.m.s. between the two independent measurements confirms the validity for both methods and the robustness of the stitching algorithm.

#### 2.2.3. Measurements of the VLS grating

The validated stitching procedure was applied for measurements of the VLS grating. The grating was rotated in the vicinity of the Littrow angle and small patches of the wavefront within the central fringe were used for the stitching to reduce the influence of retrace errors. Again, the differential wavefront error map was obtained as a difference of two measurements performed in the Littrow geometry for ±1st diffraction orders to eliminate substrate curvature effects. The local Littrow angle, α(*x*), was obtained by differentiation of the wavefront, and then the groove density along the grating length, *g*(*x*), was computed. Since such measurements do not provide an absolute value of the diffraction angles, we assumed that the groove density in the center of the pattern corresponded to the specification, *g*(0) = *g*_0_ = 178.96 lines mm^−1^, and the Littrow angle in the center was α(0) = 3.246°. VLS grating parameters, *g*_*n*_, found by the second-order polynomial fitting of the raw *g*(*x*) data, are listed in Table 2 ▸. One can see that the *g*_*n*_ coefficients are well within the specification tolerances listed in Table 1 ▸.

**Figure 7:**
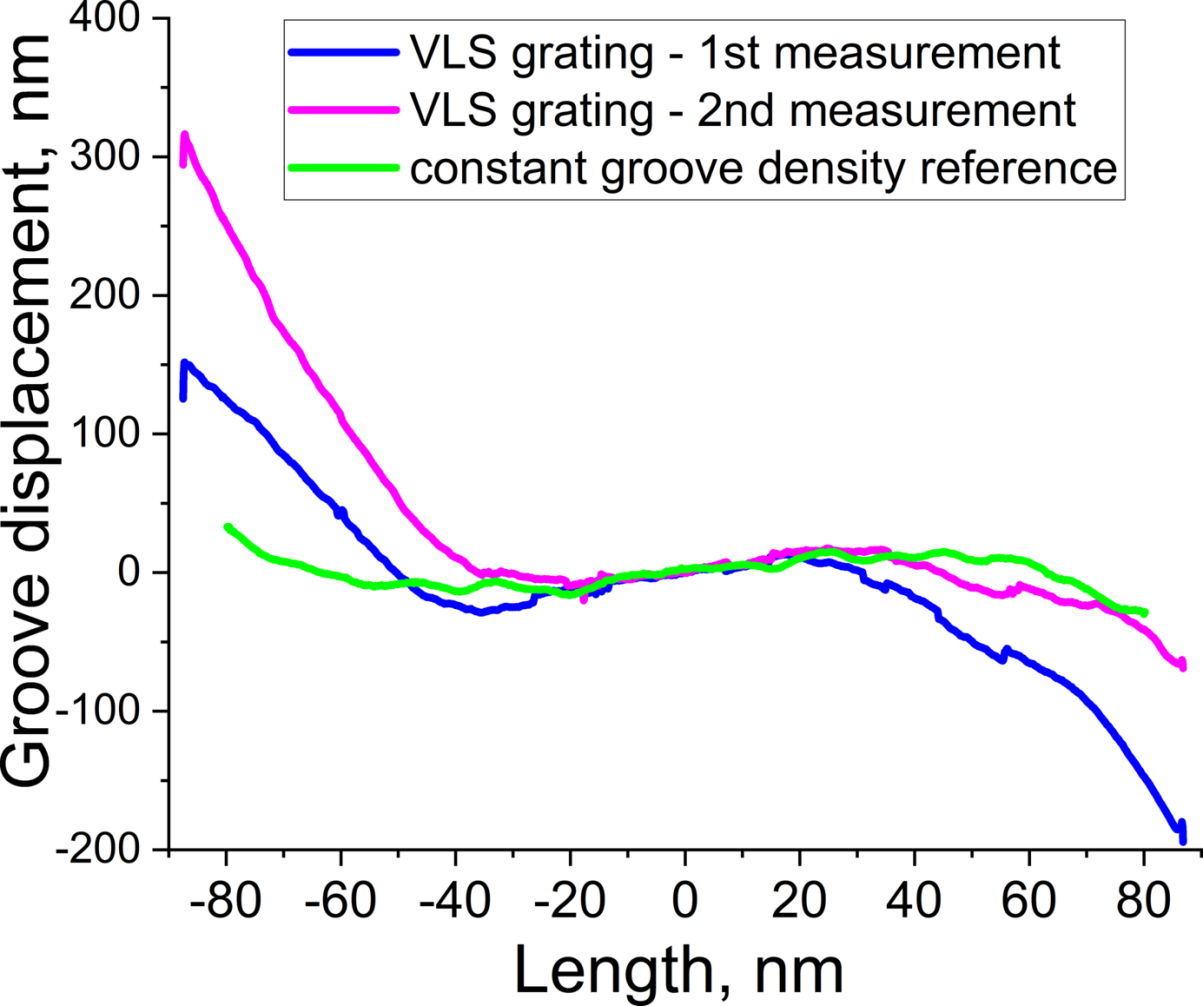
Residual groove placement errors measured for the VLS and the reference gratings.

The experimental *g*(*x*) was used to evaluate the residual groove placement errors compared with an ideal VLS grating. The local groove number, *N*(*x*), at a certain location, *x*, along the VLS pattern was found for the ideal and the experimental groove density as follows,
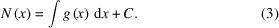
The constant *C* was found from the condition *N*(0) = 0, assigning the zeroth groove in the center of the VLS grating to be the origin. The groove displacement, Δ*X*, from an ideal position can be found as
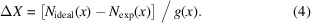
We found that a tiny uncertainty in the position of the center of the VLS pattern and the pixel size affected the accuracy of the fitted *g*_*n*_ coefficients. Although the VLS parameters obtained by fitting of the raw interferometry data and listed in Table 2 ▸ are well within the specification tolerances listed in Table 1 ▸, they result in apparent groove placement errors as large as tens of micrometres. So large errors are not consisted with the errors of ±30 nm measured for the reference pattern. Since both reference and VLS patterns were recorded in the same process one can expect that the groove placement errors are the same or very similar for both patterns. To minimize the apparent groove placement errors the fitting can be refined by adjustments of the pattern center position by 0.35 mm (*i.e.* by about 2 pixels), and the pixel size calibration by a factor of 1.000335 (by about +50 nm). These errors are within what we expect for the present system. The refined parameters of the VLS groove density are listed in Table 2 ▸. The VLS pattern was measured twice and the residual groove placement errors are shown in Fig. 7 ▸ for both the measurements along with the errors measured earlier for the reference pattern. Since the VLS pattern was created at the same time as the constant line spacing pattern, it is likely that the errors we see in Fig. 7 ▸ for the VLS case are measurement errors. This is also indicated by the 150 nm error in the reproducibility of the two nominally identical measurements. There is probably a combination of factors that produce this error, including the cumulative effect of stitching errors as a result of stitching multiple patches of the highly curved diffraction wavefront of the VLS grating. It is clear that due to these errors the reference constant groove density pattern provides a more accurate groove placement error evaluation and should be implemented in the most demanding cases. Nevertheless, the achieved accuracy of the VLS groove density measurements fully satisfies the specification requirements.

### 2.3. Diffraction efficiency measurements and simulations

**Figure 8:**
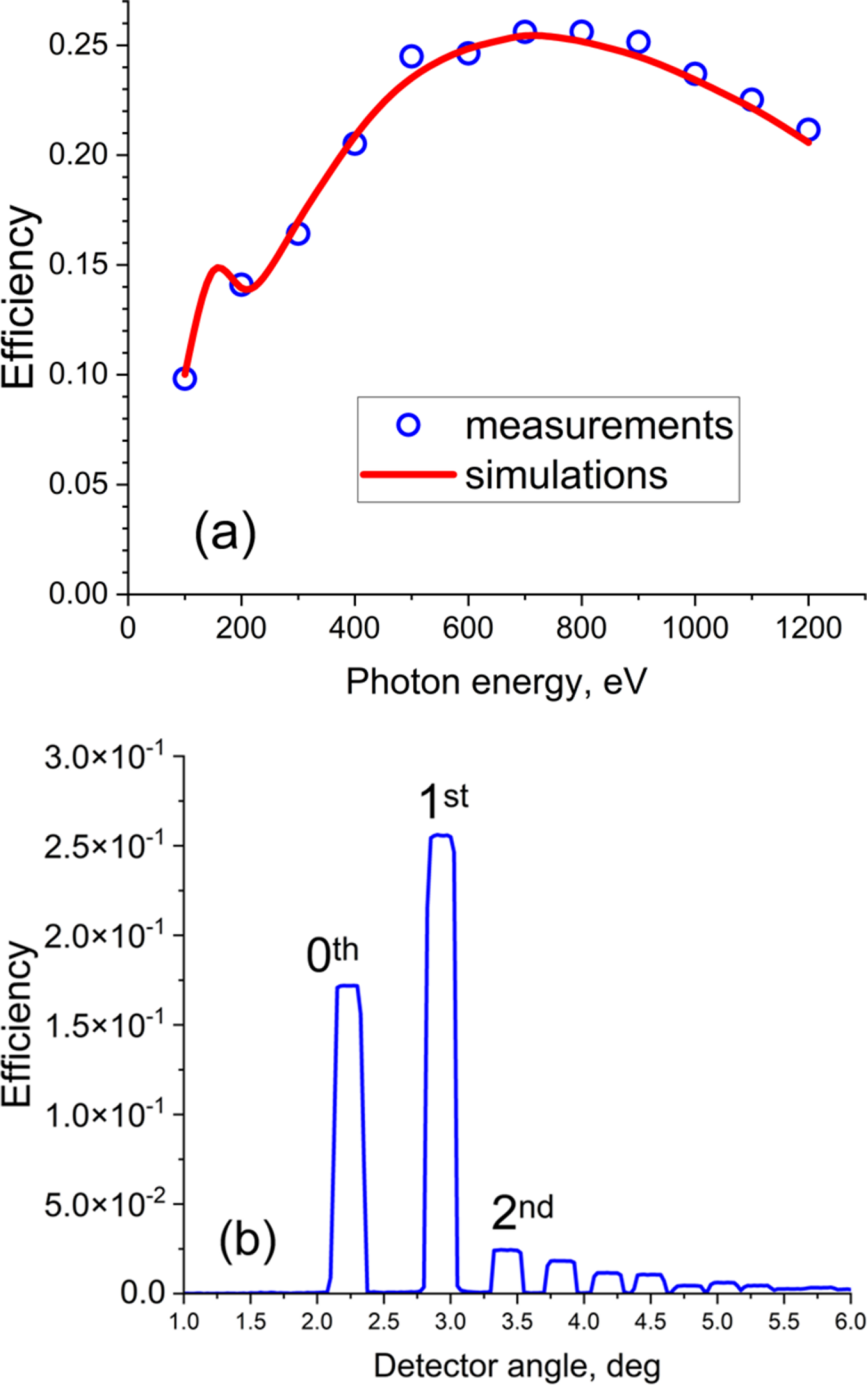
(*a*) Experimental (symbols) and simulated (curve) efficiency of the 1st diffraction order of the Au coated grating. The simulations were performed for a groove depth of 18.3 nm and land-to-pitch ratio of 0.33 measured in the center of the grating. (*b*) Detector scan at an energy of 700 eV.

The grating was coated with a 30 nm thick Au coating with a 5 nm thick Cr bonding layer deposited by DC-magnetron sputtering using Ar sputtering gas. The at-wavelength diffraction efficiency of grating #1 was measured at beamline 6.3.2 of the ALS (https://cxro.lbl.gov//reflectometer). The efficiency measurements were performed in the center of the grating by angular scans of the detector over diffraction orders. The incidence angle was set to keep the constant focusing parameter *C*_ff_ = 1.632 in the energy range 100–1200 eV (Fig. 8 ▸). Theoretical diffraction efficiency was calculated using the rigorous coupled wave analysis method using the *Reticolo* package (Hugonin & Lalanne, 2025 ▸). The measured efficiency of the first diffraction order is in good agreement with the simulation performed for the groove depth of 18.3 nm and land-to-pitch ratio of 0.33 measured in the center of the VLS pattern.

## 3. Summary

We developed a nanofabrication process for making ultra-precise lamellar X-ray gratings and a methodology for evaluation of groove placement accuracy of VLS gratings using stitching interferometry. We demonstrated the feasibility of the nanofabrication approach by fabrication of a full-size prototype of a grating for the Cosmic beamline monochromator for ALS-U. The accuracy of the VLS parameters is well within the specification and far beyond the limit achievable for holographic gratings. The grating exhibited sub-nanometre accuracy of the groove depth, a near perfect land-to-groove ratio, groove depth uniformity within 1 nm, and theoretical diffraction efficiency. Although stitching interferometry in Littrow mode agrees well with full aperture normal Littrow interferometry for constant groove density gratings, we have substantial measurement errors for VLS gratings due to strong wavefront curvature. The measurements errors are low enough for most applications, but in cases of ultra-high resolution the method should be refined to reduce these errors. In addition, for these more demanding cases we will use an adjacent constant line space grating as a reference.

## Figures and Tables

**Table 1:** VLS parameters and tolerances for the VLS groove density of the Cosmic ALS-U grating

Grating parameter	Units	Value	Tolerances
*g*_0_	mm^−1^	178.96	0.358
*g*_1_	mm^−2^	0.084000	0.00084
*g*_2_	mm^−3^	0.000014	0.0000014
Groove depth	nm	19	±2
Land-to-pitch ratio	%	38	±10

**Table 2:** VLS parameters measured by stitching interferometry for grating #2

Grating parameter	Units	Raw data fitting	After refining
*g*_0_	mm^−1^	178.93048 ± 4.4 × 10^−5^	178.9600 ± 4.43 × 10^−5^
*g*_1_	mm^−2^	0.08404 ± 5.9 × 10^−7^	0.08400 ± 5.869 × 10^−7^
*g*_2_	mm^−3^	1.416 × 10^−5^ ± 1.3 × 10^−8^	1.414 × 10^−5^ ± 1.3 × 10^−8^

## Data Availability

The data supporting the results might be available upon request.
